# New and little known Latindiinae (Blattodea, Corydiidae) from China, with discussion of the Asian genera and species

**DOI:** 10.3897/zookeys.867.35991

**Published:** 2019-07-29

**Authors:** Lu Qiu, Zong-Qing Wang, Yan-Li Che

**Affiliations:** 1 Institute of Entomology, College of Plant Protection, Southwest University, Chongqing, China Southwest University Chongqing China

**Keywords:** *
Brachylatindia
*, *
Beybienkonus
*, *
Homopteroidea
*, *
Ipolatta
*, *
Ctenoneura
*

## Abstract

A new Latindiinae, *Brachylatindia xui***gen. et sp. nov.**, is described and illustrated from Tibet, China. The new genus *Beybienkonus***gen. nov.** is established to include *Beybienkonus acuticercus* (Bey-Bienko, 1957), **comb. nov.** The Asian Latindiinae is discussed with a total of six genera included. A checklist of Asian species and a key to the Asian genera of Latindiinae are provided.

## Introduction

In the Orthopteran catalogue (Princis 1963), twelve genera (*Latindia* Stål, 1860, *Paralatindia* Saussure, 1868, *Ipisoma* Bolívar, 1893, *Ctenoneura* Hanitsch, 1925, *Compsodes* Hebard, 1917, *Homopteroidea* Shelford, 1906, *Melestora* Stål, 1860, *Bucolion* Rehn, 1932, *Biolleya* Saussure, 1897, *Buboblatta* Hebard, 1920, *Ipolatta* Karny, 1914, *Stenoblatta* Walker, 1868) were included in the family Latindiidae (now Latindiinae), two of which (*Biolleya* and *Stenoblatta*) were later transferred to the Blaberidae (Roth 2003). For the remaining genera, Roth (2003) did not specifically list any of them in Latindiinae. Only two of the remaining ten genera were regarded as Latindiinae, viz. *Latindia* and *Buboblatta*, while the remaining eight were treated as subfamily unsettled genera (Beccaloni 2014). None of the subsequent papers treated these remaining genera as Latindiinae, although all acknowledged that some of these genera may be truly related to Latindiinae (Gutiérrez 2012; Djernæs et al. 2015; Qiu, Che and Wang 2016; Wang et al. 2017). Except for problems at the generic level, the status of Latindiinae was also inconclusive. In their recent phylogenetic papers on Blattodea, both Djernæs et al. (2015) and Wang et al. (2017) indicated that Latindiinae may be upgraded to the family Latindiidae.

Three Asian genera were historically included in Latindiinae (Princis 1963), but were later excluded, i.e., *Homopteroidea*, *Ctenoneura*, and *Ipolatta*. Members of *Ctenoneura* are unique among cockroaches for their absence of the genital hook, and differ from Latindiinae by the asymmetrical subgenital plate, single stylus, more complex venation, and the apterous female (Qiu et al. 2017). However, *Homopteroidea* and *Ipolatta* were excluded from Latindiinae without providing any reason; recent papers now indicate that the Asian Latindiinae are more diverse than previously thought: Qiu et al. (2016) reported the genus *Sinolatindia* from China and Lucañas (2018) described genus *Gapudipentax* from the Philippines.

Since Latindiinae species are small and unnoticeable, specimens are difficult to obtain. Recently, we obtained some living individuals and specimens from Yunnan and Tibet, China. All materials were collected from the rotten wood. We thus take this opportunity to carefully study them and report upon this little known subfamily from China. The status of *Homopteroidea* and *Ipolatta* are reconsidered, and a checklist of the Latindiinae species from Asia as well as a key to the Asian genera are provided.

## Materials and methods

The specimens from China are all deposited in the Institute of Entomology, Southwest University, Chongqing, China (**SWU**). We also examined specimens of the *Homopteroidea* collection in Oxford University Museum of Natural History, Oxford, UK (**OUM**). Those specimens include the lectotype and two paralectotypes of *Homopteroidea shelfordi* (ORTH0206 1/4, 3/4–4/4), the paratype of *Ctenoneura aberrans* (= *Homopteroidea aberrans*) (ORTH342 2/2), the holotype of *Homopteroidea maculate* (ORTH363), the lectotype of *Homopteroidea minor* (ORTH389 1/2), and slides studied by Roth (1995a), viz. slide 272 (tegmina and a wing), slide 273 (male genitalia and subgenital plate) of *Homopteroidea nigra*, and slide 271 (a tegmen), and slide 278 (male genitalia) of *Homopteroidea brachyptera*.

The definition of Latindiinae here follows that of Qiu et al. (2016) and Wang et al. (2017). Morphological terminology used in this paper mainly follows Roth (2003), genitalia terms follow Klass (1997), and venation terms follow Li et al. (2018).

The genital segments of the examined specimens were dipped in 10% NaOH and observed in glycerine jelly using a Motic K400 stereomicroscope and a Leica M205A stereomicroscope. Venation drawings were made with the aid of Adobe Photoshop CS6, a Leica M205A stereomicroscope and a Motic K400 stereomicroscope. Photographs of the habitus and characters were made using a Leica M205A stereomicroscope. All photographs were modified in Adobe Photoshop CS6.

For pairing and comparison, we selected seven samples representing different morphologies and genders from different locations to sequence their COI genes. The COI sequences are deposited in the National Center for Biotechnology Information GenBank (accession numbers MN116495, MN116496, MN116497, MN116498, MN116499, MN116500, MN116501). The extraction procedure was according to the Hipure Tissue DAN Mini Kit. Total DNA was stored at -20 °C. Primers for the amplifications are COI-F3 (5’-CAACYAATCATAAAGANATTGGAAC-3’) and COI-R3 (5’-TAAACTTCTGGRTGACCAAARAATCA-3’). The amplification conditions were as follows: initial denaturation at 98 °C for 2 min, followed by 35 cycles of 10s at 98 °C, 10s for 51 °C, and 15s for 72 °C, with final extension of 2 min at 72 °C. Laboratory reagents were provided by TsingKe Co, Ltd., China. All voucher specimens are deposited at SWU. The genetic divergence value was quantified based on the Kimura 2-parameter (K2P) distance model (Kimura 1980), using MEGA 7 (Tamura et al. 2013) with 1000 bootstrap replicates.

## Taxonomy

### Brachylatindia gen. nov.

Taxon classificationAnimaliaBlattodeaCorydiidae

b11ac20d-5c3c-566f-b45d-8790575652d7

http://zoobank.org/8A2734A8-F5DD-4133-955C-8749BE50AA14

#### Type species.

Here designated: *Brachylatindia xui* sp. nov.

#### Diagnosis.

Small, brachypterous, smooth but with sparse micro spines. Head oval, ocelli absent; pronotum roundly triangular, meso- and meta- notum somewhat reduced; front femur type C_2_, tarsal claws simple, arolia present; male with a gland at the centre of 4^th^ tergum; subgenital plate of female valved.

This new genus resembles *Gapudipentax* Lucañas, 2018, but it can be readily distinguished from the latter by the following characters: 1) head sub-oval, while head triangular in *Gapudipentax*; 2) pronotum triangular with rounded edges, smooth, with indistinct micro setae, while pronotum pentagonal with rounded edges, distinct pubescent in *Gapudipentax*; 3) male with subtriangular tegmen, while male with subquadrate tegmen in *Gapudipentax*; 4) front femur with two long apical spine at hind margin, while front femur without any long apical spines in *Gapudipentax*; 5) tarsal claw not serrated, while tarsal claw serrated in *Gapudipentax*; and 6) male with tergal gland, while male without tergal gland in *Gapudipentax*.

#### Generic description.

Body small, smooth, sexual dimorphism indistinct, both brachypterous. Male: head longer than width, oval, vertex not exposed. Ocelli absent. Pronotum triangular with rounded edges, with indistinct and micro setae. Meso- and meta- notum reduced, narrowed, median of both slightly extended. Tegmina reduced, reaching only up to the middle of the 2^nd^ tergum; wing reduced, very small (flightless). Front femur type C_2_, apex without spine; mid- and hind femora each with a spine at apex and a spine at apical portion of hind margin. Tarsomere 1 longer than the rest of tarsomeres combined. Pulvulli absent. Tarsal claws simple, symmetrical. Arolia present. Abdomen with 4^th^ tergum specialised, with a gland medially. Supra-anal plate trapezoidal, with large hyaline area, apex concave; paraprocts hooked at apical portions; cerci each with a small spine at apex. Subgenital plate symmetrical; styli simple, similar. Genitalia complex, with long and robust genital hook (L3), R2 elongate.

Female: similar to male. Tegmina reduced. Metanotum normal, wings absent. Supra-anal plate subtriangular, apex emarginated. Subgenital plate valved, medial slit entire, through the apex to the base.

#### Geographical distribution.

China (Tibet).

#### Etymology.

*Brachys* (Greek for short) + *latindia* refers to a Latindiinae cockroach with brachypterous tegmina.

### Brachylatindia xui sp. nov.

Taxon classificationAnimaliaBlattodeaCorydiidae

4774287d-2856-5409-9d73-da3662791234

http://zoobank.org/EAF2ADE4-6D04-4179-8DA1-2BB66F34F300

**Figure 1.**
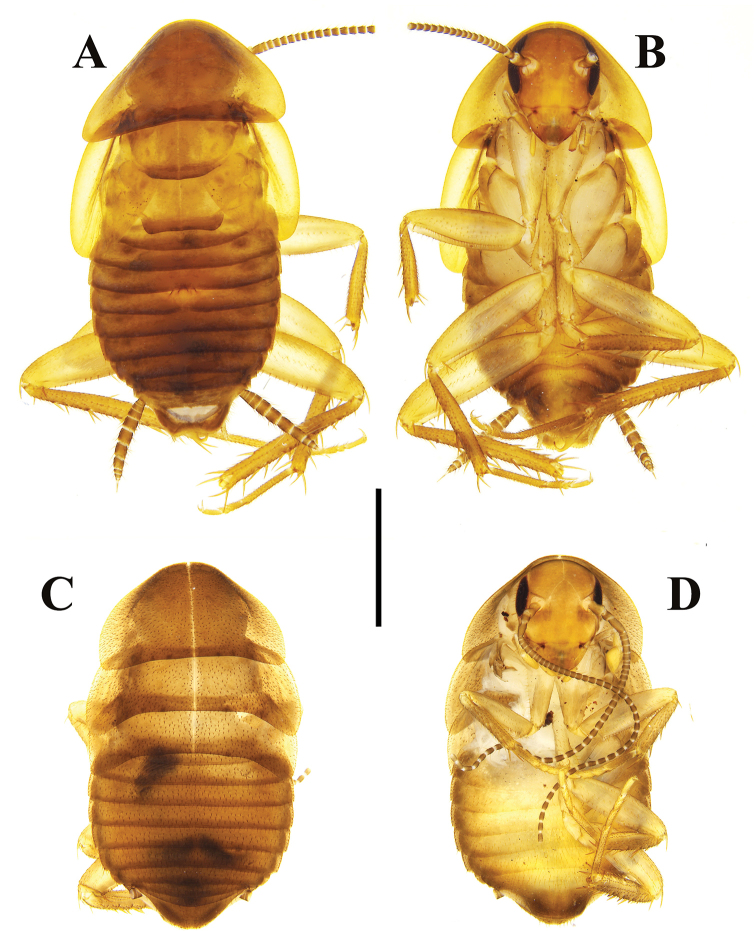
**A–D***Brachylatindia xui* gen. et sp. nov. **A** male holotype, dorsal view **B** same, ventral view **C** nymph paratype, dorsal view **D** same, ventral view. Scale bar: 2 mm.

**Figure 2.**
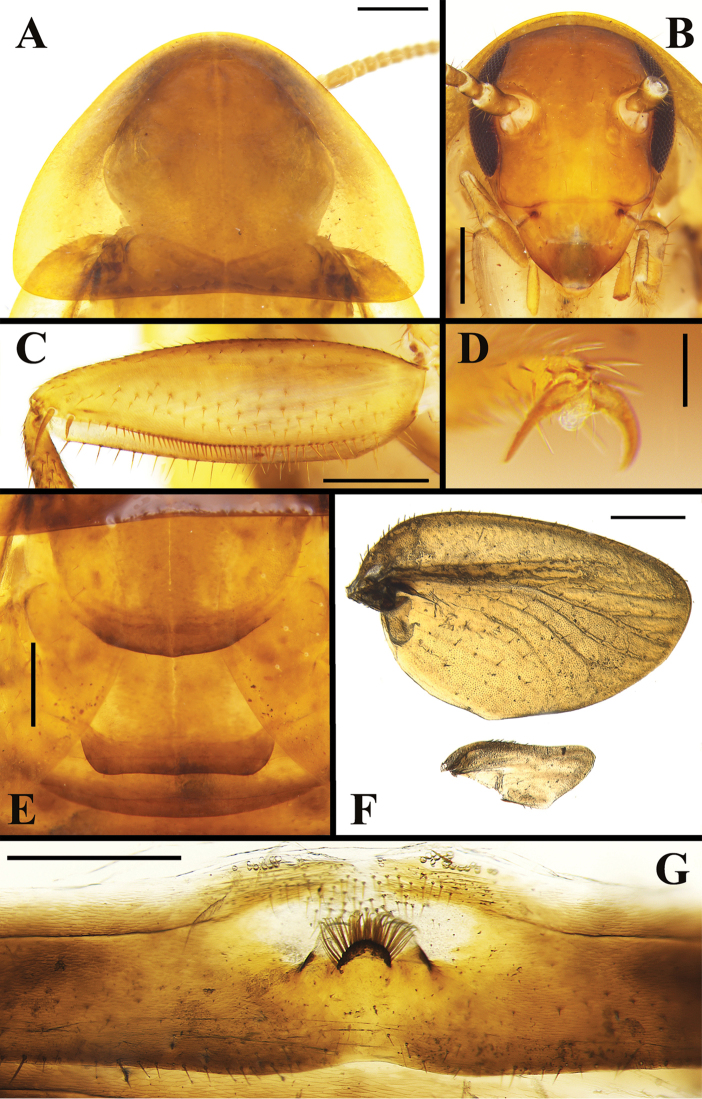
*Brachylatindia xui* gen. et sp. nov., male holotype **A** pronotum, dorsal view **B** head, ventral view **C** front femur **D** tarsal claw **E** meso-and meta- notum **F** right tegmen and wing **G** tergal modification in the 7^th^ tergum of abdomen. Scale bars: 0.5 mm (**A–C, E–G**); 0.1 mm (**D**).

**Figure 3.**
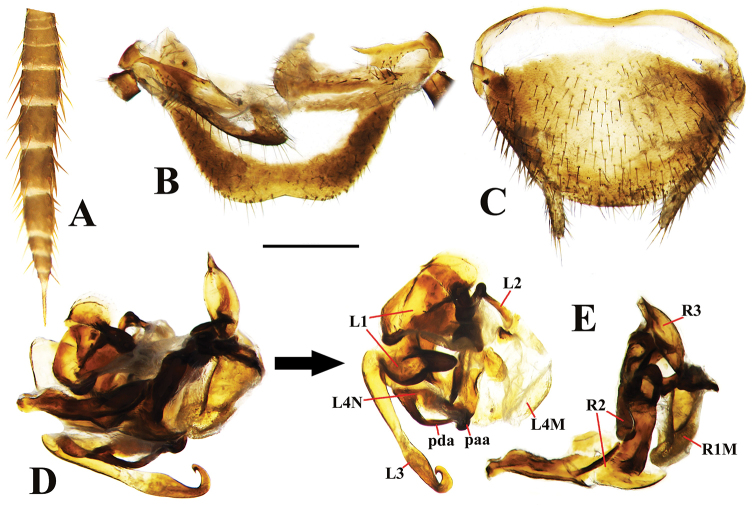
*Brachylatindia xui* gen. et sp. nov., male holotype **A** cercus **B** supra-anal plate, ventral view **C** subgenital plate, ventral view **D** genitalia, original position **E** genitalia (dissected). Scale bar: 0.5 mm.

Figs 1 , 2 , 3 , 11A

#### Type material.

**Holotype**, male (SWU): **CHINA: Tibet (= Xizang)**: Upper Zayü (= Shangchayu) Town [上察隅镇], Zayü (= Chayu) County [察隅县], Nyingchi (= Linzhi) City [林芝市], alt. 1900 m, 9.VII.2016, Hao Xu et Jian-Yue Qiu leg. **Paratype**: 1 nymph (SWU), same data as holotype.

#### Diagnosis.

As for the genus (vide supra).

#### Description.

**Male (holotype). General**: measurements (mm): body length (vertex to abdomen tip): 6.2, pronotum length × width: 2.2 × 3.0, tegmen length: 2.1. Size small, brownish yellow, tegmina and wings reduced (Fig. 1A, B). **Head**: oval, with very sparse setae, brownish yellow. Vertex convex, sheltered under pronotum. Eyes small, wide apart; interocular space much greater than the distance between antennal sockets. Ocelli absent. Frons smooth, two very shallow spots situated between the lower parts of the antennal sockets. Antennal sockets small, each with a row of setae at upper margin. Antennae dark brown, long, 7.4 mm, longer than the body length. Clypeus small, nearly trapezoidal, ante-clypeus and post-clypeus not indicated. Labrum small, sub-triangular, apex blunt. Maxillary palpi moderate (Fig. 2B). **Pronotum**: brownish yellow, lateral parts sub-transparent. Smooth, surface without pubescence, but very sparse micro setae (cannot be observed by naked eyes, but visible under microscope). Shape subtriangular, widest near the hind angles, apex rounded, hind angles rounded (Fig. 2A). **Mesonotum and metanotum.** Both somewhat reduced; mesonotum semi-oval, apical margin thickened; apical margin of metanotum protruded, almost reaching to half of the 1^st^ tergum, the protruded part quadrated and thickened (Fig. 2E). **Tegmina and wings**: both reduced, flightless. Tegmen smooth, almost reaching the edge of the 2^nd^ tergum, lobate, apex rounded; venation reduced, main veins simple. Wing small, triangular, venation indistinct (Figs 1A, 2F). **Legs**: smooth, setose, whitish yellow, tibiae and tarsi brownish yellow. Front femur with a row of small spines at hind margin, ending with a long spine and a short spine near apex (type C_2_) (Fig. 2C). In middle and hind femur, each femur with a row of sparse spines at hind margin, ending with one long spine; one long spine appearing at the apex of anterior margin. Tibiae normally with some long spines and short setae. Tarsi covered with many spines; the length of tarsus 1 longer than the total length of tarsi 2 to 5; tarsal claws normal, symmetrical, small; arolia minute (Fig. 2D). **Abdomen**: smooth, brownish, 4^th^ tergum specialised, with a gland medially, hind margin of 4^th^ tergum thinned and slightly concave in the middle (Fig. 2G). Supra-anal plate with a large transparent area medially, apex widely concave, margin setose; paraprocts with long setae; cerci long, lateral portions setose, apex with a spine (Figs 3A–B). Subgenital plate setose; styli cylindrical (Fig. 3C). **Genitalia**: complex. **Left phallomere**: L1 large, consists of two irregular sclerites, the ventral one with a stick-like process on the left, apex round; L2 small, elongate and curved, median with a lamina; L3 very robust, apical portion enlarged, then thinner and curved toward apex, apex sharp; L4N with pda and paa well developed, long and sharp; L4M thin, transparent. **Right phallomere**: R1M and R3 small; R2 with two distinct elongate sclerites, the ventral one short, stick-like, apex enlarged and rounded, the dorsal one extremely long, lying across the whole phallomere (Fig. 3D, E).

#### Female.

Unknown.

#### Nymph.

Similar to the adult, but body densely pubescent (Fig. 1C, D).

#### Ootheca.

Unknown.

#### Natural history.

Individuals were collected from the rotten wood (H. Xu et J.-Y. Qiu, pers. comm.) (Fig. 11A).

#### Distribution.

China (Tibet) (Fig. 13).

#### Etymology.

This new species is named after Dr. Hao Xu, one of the collectors of this new species, for his efforts in collecting this cockroach.

### Brachylatindia

Taxon classificationAnimaliaBlattodeaCorydiidae

sp.

0e44d6c6-c9d4-5d38-b947-d0bebc4ea246

Figs 4 , 5 , 11B

#### Material examined.

1 female (SWU), **CHINA: Tibet**: Jialongba [加龙坝], Suotong Village [索通村], Guxiang Township [古乡], Bomê (= Bomi) County [波密县], Nyingchi City [林芝市], alt. 2300 m, 23.VII.2016, Jian-Yue Qiu et Hao Xu leg.

#### Diagnosis.

**Figure 4.**
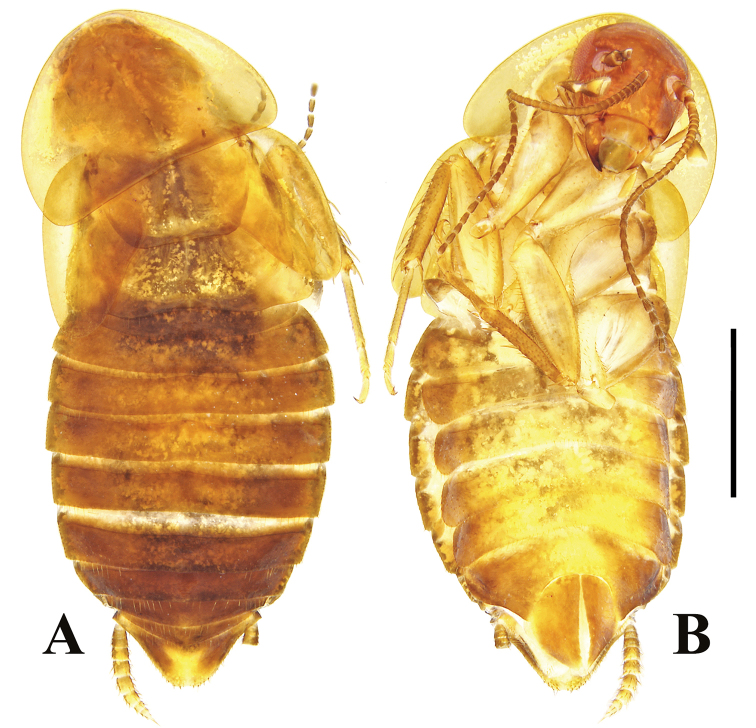
*Brachylatindia* sp., female from Tibet **A** dorsal view **B** ventral view. Scale bar: 2 mm.

**Figure 5.**
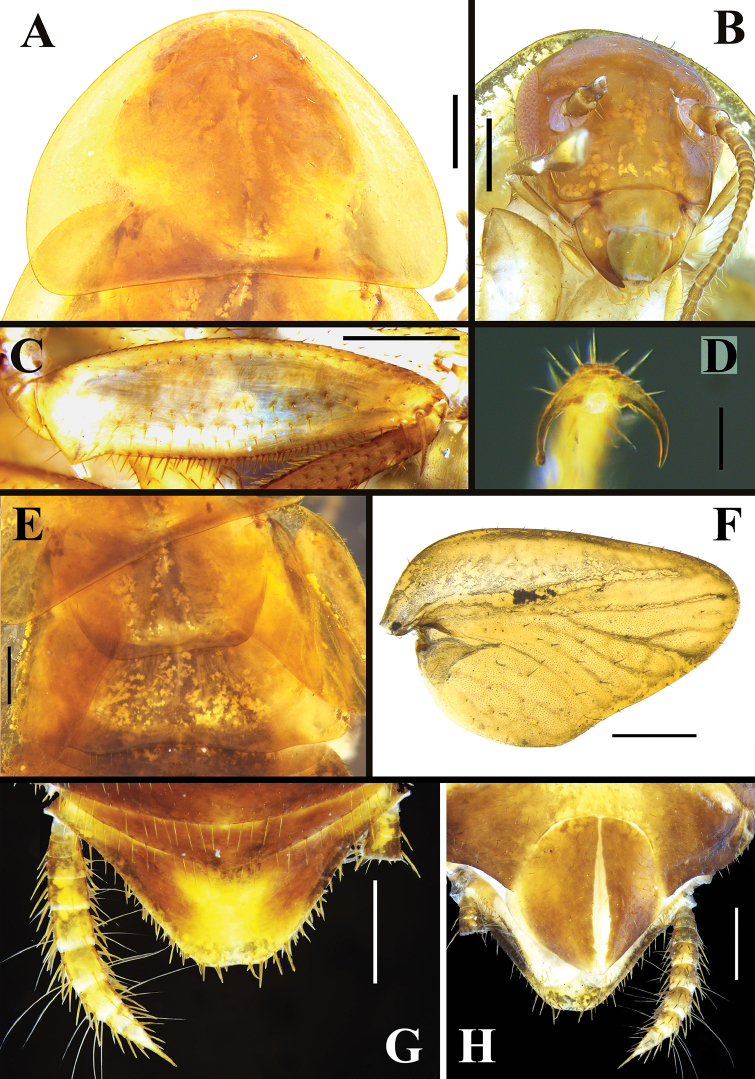
*Brachylatindia* sp., female from Tibet **A** pronotum, dorsal view **B** head, ventral view **C** front femur **D** tarsal claw **E** meso-and meta- notum **F** right tegmen **G** supra-anal plate, dorsal view **H** subgenital plate, ventral view. Scale bars: 0.5 mm(**A–C, E–H**); 0.1 mm (**D**).

Body small, brownish yellow (Fig. 4A, B). Body length (vertex to abdomen tip): 7.4 mm, pronotum length (midline) × width (the widest points): 2.3 × 3.0 mm, tegmen length: 2.1 mm. Head oval (Fig. 5B), pronotum smooth, triangular (Fig. 5A). Mesonotum reduced, trapezoidal; metanotum wide, not reduced (Fig. 5E). Tegmina short, triangular, venation simple (Fig. 5F); wings absent. Front femur type C_2_ (Fig. 5C), arolia present but small (Fig. 5D). Abdomen without tergal modification. Supra-anal plate with a yellowish area medially, apex narrowed, concave, margin setose (Fig. 5G); subgenital plate valved (Fig. 5H).

#### Natural history.

This species was collected from the rotten wood from the forest of Guxiang, Bomi (H. Xu et J.-Y. Qiu, pers. comm.) (Fig. 11B).

#### Geographical distribution.

China (Tibet) (Fig. 13).

#### Remarks.

This species is similar to the male of *Brachylatindia xui* sp. nov., but its front femur has a right-angle protrusion near the base (Fig. 5C), mesonotum is trapezoidal, metanotum is not reduced (Fig. 5E), tegmina are larger, and the wings are absent. These differences may be sexually dimorphic, so to further verify they are different species, we sequenced the COI genes of *B. xui* sp. nov. and this female specimen (GenBank access numbers MN116501 and MN116499, respectively, for the male and nymph specimens of *B. xui* sp. nov., MN116496 for this female specimen). The results show that the divergence between the two species is 15.7% (0% between the holotype and paratype of *B. xui* sp. nov.). This result indicates that this specimen is not the female of *Brachylatindia xui* sp. nov.

### Beybienkonus gen. nov.

Taxon classificationAnimaliaBlattodeaCorydiidae

e9117352-a06f-5d5e-86f0-5518a0a73b74

http://zoobank.org/2108E9F0-AFE5-41CB-95BE-C829B30DCFE4

#### Type species.

*Beybienkonus acuticercus* (Bey-Bienko, 1957) comb. nov.

#### Diagnosis.

This new genus is unique by having two robust spines on each hind femur and one curved robust spine at the apex of each hind tibia in male. The hind legs of male are robust. Apex of each cercus with a long spine in both sexes. Both brachypterous and macropterous types are present in this genus.

#### Generic description.

Body large for Latindiinae, smooth; both brachypterous and macropterous types are present.

#### Brachypterous male.

Head longer than width, oval, vertex slightly exposed. Ocelli represented as two white spots. Pronotum semi-oval, smooth. Meso- and meta- notum slightly reduced, median not extended. Tegmina reduced, reaching up to half of abdomen; wing reduced, small and elongate (flightless). Front femur type C_1_; mid- and hind femora each with a spine at apex and two spines at apical portion of hind margin, the two spines of hind femur well developed, robust. Hind tibia with a robust long spine and a thin long spine at apex. Tarsomere 1 longer than the rest of tarsomeres combined. Pulvulli absent. Tarsal claws simple, symmetrical; arolia absent. Abdomen without tergal modification. Supra-anal plate narrowly triangular, with large hyaline area medially, apex with two rounded lobes; paraprocts simple; cerci smooth dorsad, setose ventrad, each with a long spine at apex. Subgenital plate symmetrical; styli simple, similar. Genitalia with small genital hook (L3), right phallomere large.

#### Macropterous male.

Unknown.

#### Brachypterous female.

Similar to brachypterous male. Tegmina more reduced, not exceeding the half of abdomen, wing much more reduced, very small (flightless). Hind legs not as robust as male, spines normal, not enlarged. Supra-anal plate subtriangular, apex emarginated, margin setose, ventral surface setose. Subgenital plate valved, medial slit entire through the apex to the base.

#### Macropterous female.

Body relatively narrowed. Pronotum oval, small. Tegmina and wings fully developed exceeding the end of abdomen (capable of flight). The remaining characters similar to the brachypterous female.

#### Geographical distribution.

China (Yunnan and Tibet).

#### Etymology.

Named after Bey-Bienko G.Y., the Russian entomologist, who first reported the type species *Beybienkonus acuticercus* (Bey-Bienko, 1957) comb. nov. from Yunnan, China.

### Beybienkonus acuticercus

Taxon classificationAnimaliaBlattodeaCorydiidae

(Bey-Bienko, 1957) comb. nov.

8484df6c-9d4d-5d68-bf40-57ac93142903

**Figure 6.**
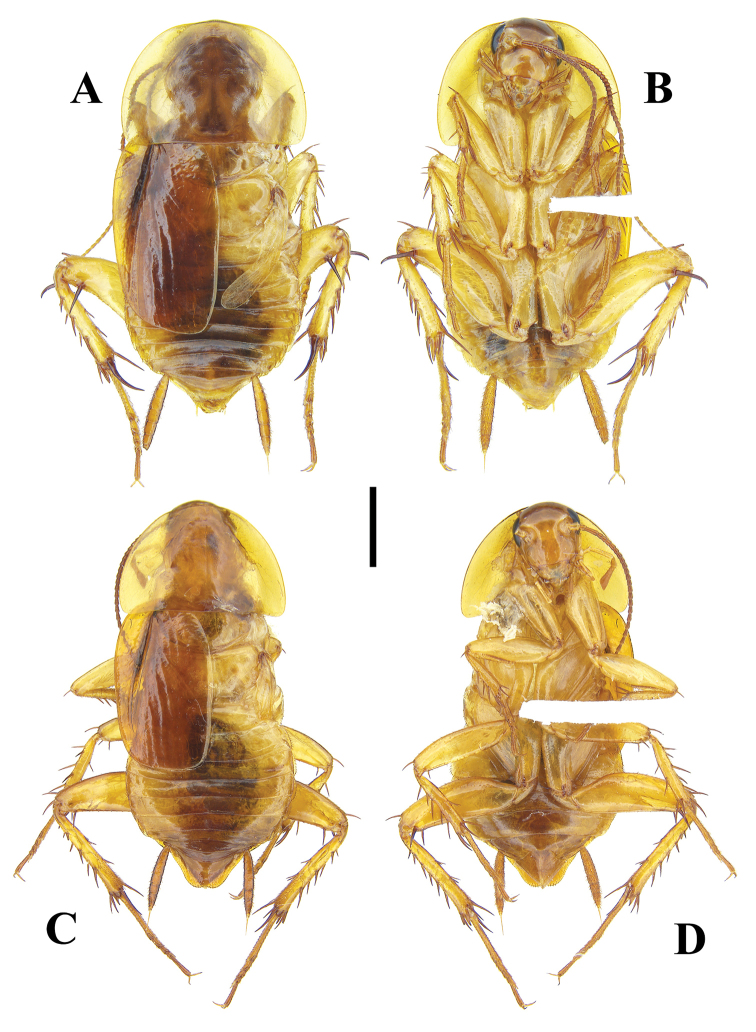
**A–D***Beybienkonus acuticercus* (Bey-Bienko, 1957), comb. nov., brachypterous individuals from Yunnan **A** male, dorsal view **B** same, ventral view **C** female, dorsal view **D** same, ventral view. Scale bar: 2 mm.

**Figure 7.**
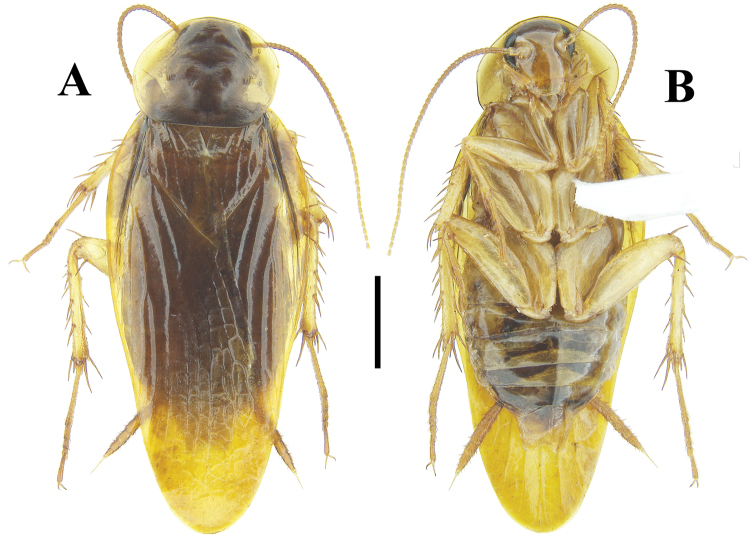
**A–B***Beybienkonus acuticercus* (Bey-Bienko, 1957), comb. nov., macropterous female from Yunnan **A** dorsal view **B** ventral view. Scale bar: 2 mm.

**Figure 8.**
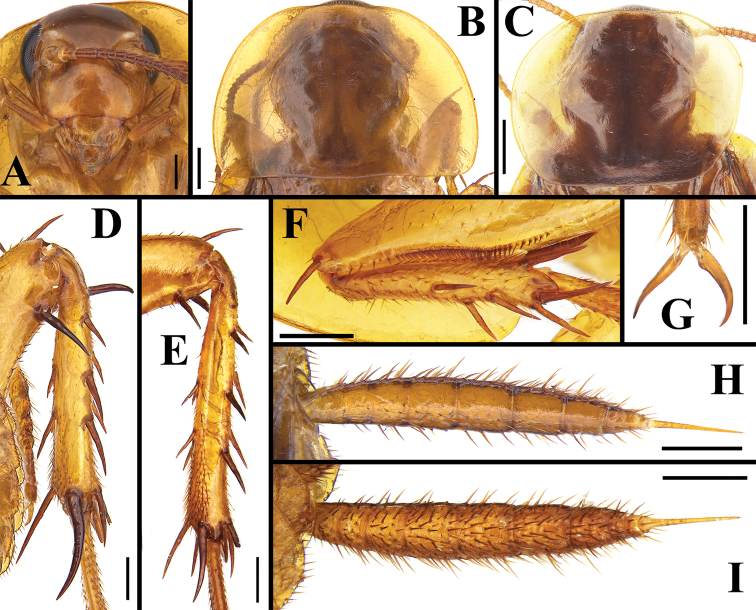
*Beybienkonus acuticercus* (Bey-Bienko, 1957), comb. nov., individuals from Yunnan **A** head, brachypterous male, ventral view **B** pronotum, brachypterous male, dorsal view **C** pronotum, macropterous female, dorsal view **D** part of the hind leg, male **E** part of the hind leg, female **F** front femur, male **G** tarsal claw, male **H** cercus, male, dorsal view **I** cercus, male, ventral view. Scale bars: 0.5 mm.

**Figure 9.**
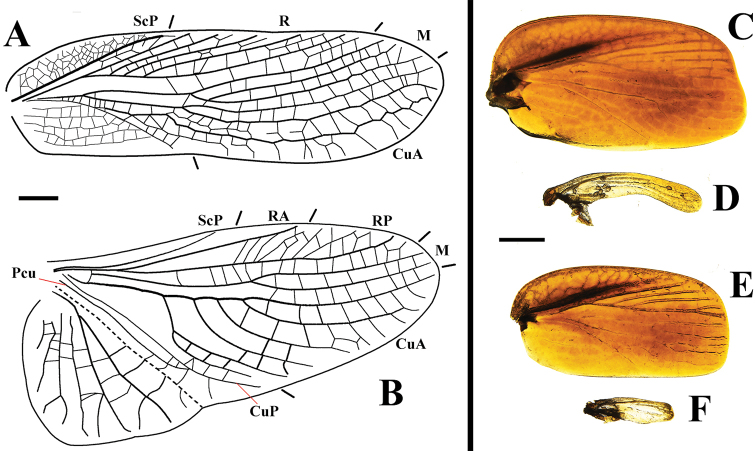
Tegmina and wings of *Beybienkonus acuticercus* (Bey-Bienko, 1957), comb. nov., individuals from Yunnan **A** tegmen, macropterous female **B** wing, macropterous female **C** tegmen, brachypterous male **D** wing, brachypterous male **E** tegmen, brachypterous female **F** wing, brachypterous female. Scale bars: 0.5 mm.

**Figure 10.**
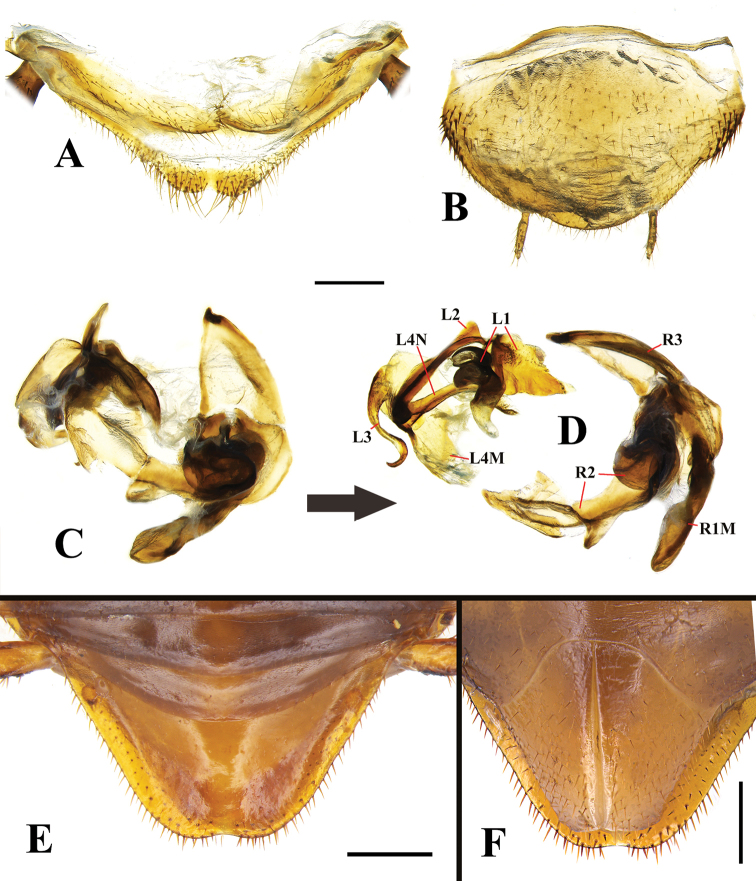
*Beybienkonus acuticercus* (Bey-Bienko, 1957), comb. nov., individuals from Yunnan **A** supra-anal plate, male, ventral view **B** subgenital plate, male, ventral view **C** genitalia, male, original position **D** genitalia, male (dissected) **E** supra-anal plate, female, dorsal view **F** subgenital plate, female, ventral view. Scale bars: 0.5 mm.

**Figure 11.**
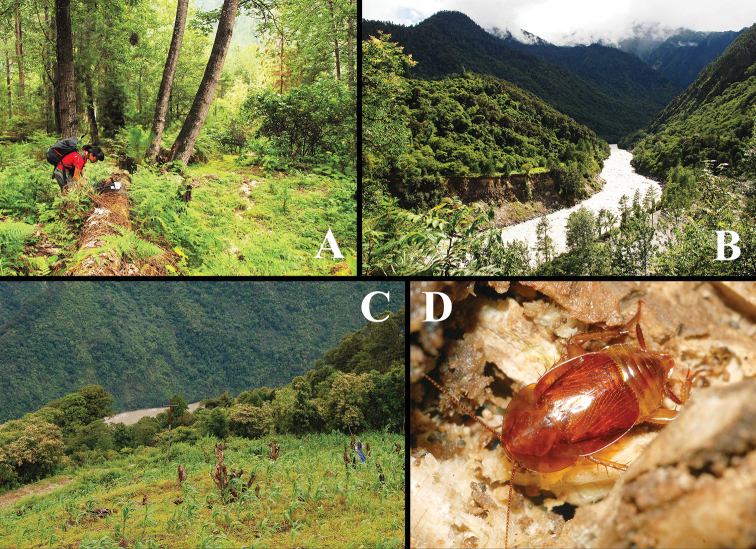
Habitats of Latindiinae from China **A** habitat of *Brachylatindia xui* gen. et sp. nov., Shangchayu, Chayu, Tibet **B** habitat of *Brachylatindia* sp., Guxiang, Bomi, Tibet **C–D** habitat of *Beybienkonus acuticercus* (Bey-Bienko, 1957), comb. nov., Motuo, Tibet **D** a living *B. acuticercus* (Bey-Bienko, 1957), comb. nov., found in rotten wood. All photographs by Hao Xu.

Figs 6 , 7 , 8 , 9 , 10 , 11C–F

Ctenoneura acuticerca Bey-Bienko, 1957: 896 (original description); Princis 1963: 101 (catalogue); Roth 1993: 87; Feng et al. 1997: 34 (catalogue); Qiu et al. 2017: 297.

#### Material examined.

**CHINA: Yunnan**: 5 males (brachypterous), 3 females (macropterous), 10 females (brachypterous), and more than 30 nymphs (under rearing): around Mangyun Township [芒允乡], Yingjiang County [盈江县], Dehong Prefecture [德宏景颇族自治州], 24°34'N, 97°45'E, alt. ca. 800–1300 m, 27.II–11.III.2018, Gui-Chang Liu (local people) leg.; **Tibet**: 3 females (brachypterous): Gelin Village [格林村], Bengbeng Township [背崩乡], Medog County [墨脱县], Nyingchi City [林芝市], alt. 1600 m, 15.VII.2016, Hao Xu et Jian-Yue Qiu leg. (all in SWU).

#### Description.

**Brachypterous male. General**: measurements (mm): body length (vertex to abdomen tip): 10.1–10.6, pronotum length (midline) × width (the widest points): 3.1–3.2 × 4.6–4.8, tegmen length: 4.9–5.1, tegmen width: 2.7–2.8. Size small, body smooth, brownish yellow (Fig. 6 A, B). **Head**: longer than width. Vertex slightly exposed under pronotum, convex, darker than the remaining part of head. Eyes small, wide apart; interocular space much greater than the distance between ocelli and antennal sockets. Ocelli represented as two white spots, situated above antennal sockets. Frons smooth, two brown spots situated between the lower parts of the antennal sockets. Antennal sockets small. Antennae dark brown, shorter than the body length (8.6–10.3 mm). Face smooth, large. Clypeus small, nearly trapezoidal, the edge between ante-clypeus and post-clypeus indistinct. Labrum small, sub-triangular. Maxillary palpi long (Fig. 8A). **Pronotum**: light brownish yellow, lateral parts sub-transparent. Smooth, surface without pubescence, but very sparsely with micro setae (can’t be observed by naked eyes, even easily overlooked under microscope). Shape semi-oval, widest at 1/4 from the base, hind angles round (Fig. 8B). **Tegmina and wings**: both reduced, flightless. Tegmen smooth, brown, reaches to the 4^th^ or 5^th^ tergum, nearly rectangular, apical portion slightly protruding, overall outline slightly rounded; venation simple. Wing small, elongate, curved, reaching the apex of 2^nd^ or 3^rd^ tergum (Figs 6A, 9C, D). **Legs**: smooth, brownish yellow, sparsely covered with short setae, hind legs robust. Front femur with a row of small spines at hind margin, ending with a long spine at apex (type C_1_) (Fig. 8F), while in middle and hind femur without row of spines at hind margin; in middle and hind legs, each femur with a long spine at anterior apex and two long spines at hind apex; in hind femur, the two spines at hind apex extremely robust and long. Tibia with a spinous protrusion at apex (small in front and middle tibiae, extremely long in hind tibia); surface of tibia normally with some long spines, the spines at tibial apex longer than the spines at tibial surface; in hind tibia, two of the apical spines extremely long (one is thin and straight, the other is robust and curved) (Fig. 8D). Tarsi covered with many spines; the length of tarsus 1 sub-equal to the total length of tarsus 2 to 5; tarsal claws normal, symmetrical, moderate in size; arolia absent (Fig. 8G). **Abdomen**: smooth, brownish, terga without modification, lateral margins with small spinous pubescence. Supra-anal plate (Fig. 10A) pubescent, narrowly triangular, apex with two rounded lobes; paraprocts simple; cerci long, smooth and without pubescence dorsally, with pubescent ventrally, apex with a very long and sharp spine (Fig. 8H, I). Subgenital plate simple, sparsely pubescent, base with rough setae laterally; styli slender (Fig. 10B). **Genitalia: Left phallomere**: L1 consists of two irregular sclerites, the dorsal one slice-like, the ventral one with three unequal-sized protrusions; L2 thick, straight; L3 small, curved, S-shaped; L4N simple, straight; L4M small, slice-like. **Right phallomere**: large. R3 and R1M elongate; R2 with two sclerites, the ventral one stout, irregularly rounded, the dorsal one extremely long, irregular, lays across the whole phallomere (Figs 10C–D).

#### Brachypterous female.

Measurements (mm): body length: 8.9–10.5, pronotum length × width: 3.0–3.2 × 4.5–4.7, tegmen length: 4.4–4.7, tegmen width: 2.5–2.7. Generally similar to the brachypterous male, but eyes slightly smaller than that of the male, antennae shorter than the body length (Fig. 6C, D). Tegmen shorter, apex truncated, only reaching half of the 3^rd^ or 4^th^ tergum; wing much more reduced, only reaching the 1^st^ tergum; venation simple (Fig. 9E, F). Spines on legs normal, not elongated or enlarged (Fig. 8E). Supra-anal plate trapezoid-shaped, apex rounded, median slightly concaved, margin and ventral surface setose (Fig. 10E). Subgenital plate valved (Fig. 10F).

#### Macropterous male.

Unknown.

#### Macropterous female.

Measurements (mm): body length: 9.2–9.4, total length: 10.9–12.5, pronotum length × width: 2.3–2.6 × 3.2–3.6, tegmen length: 8.9–10.1, tegmen width: 2.9–3.1 (Figs 7A–B). Head and legs the same as the brachypterous females. Pronotum sub-oval, wider than long, hind margin slightly truncated (Fig. 8C). Tegmina and wings fully developed. Tegmen with a thick and simple ScP, ScP area articulated with many small veins; R with four branches; M with two long branches that are parallel with the main vein; CuA bifurcated at basal half, near the middle, an isolated vein present, with many articulated cross veins connecting it with the main vein of CuA. Wing with a single ScP and RA; RP simple, with many thin veins; M bifurcated medially; CuA with four curved and parallel branches; CuP and Pcu simple (Fig. 9A, B). Abdomen slightly narrower than the brachypterous female.

#### Nymph.

**Figure 12.**
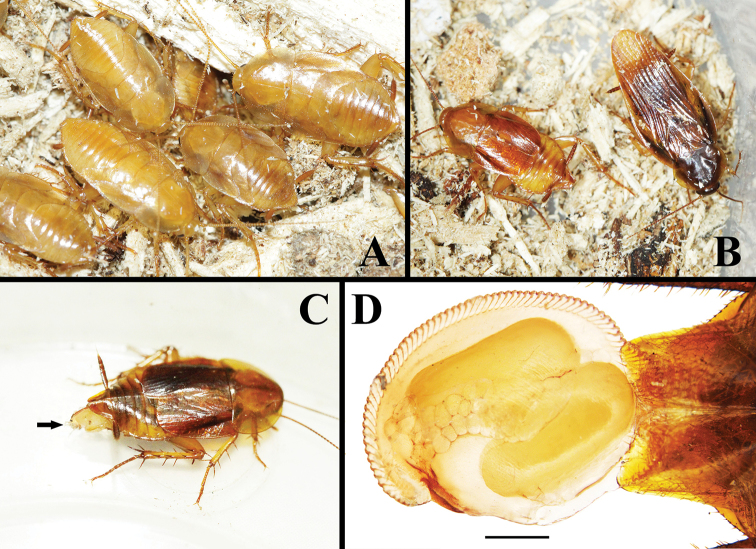
*Beybienkonus acuticercus* (Bey-Bienko, 1957), comb. nov. from Yingjiang, Yunnan, under lab conditions **A** nymphs **B** macropterous and brachypterous females. **C** brachypterous female with ootheca (black arrow indicated) **D** ootheca. Scale bar: 0.5 mm. All photographs by Lu Qiu.

Large nymphs light brownish yellow, sub-transparent, densely pubescent (Fig. 12A).

#### Ootheca.

Flat, rounded, with only two eggs, dense serrations present at the keel (Fig. 12D).

#### Natural history.

Individuals were captured from rotten wood, or under the barks of the rotten wood (Fig. 11C, D). Under the lab condition, individuals can feed on bread crumbs and apple pieces; one can prevent the others from grabbing its food by kicking (by the strong hind legs), or fast running away with food (food were carried by front legs). Females were noticed producing oothecae in April (Fig. 12C), the nymphs were very fast hatched around 10–15 days.

#### Distribution.

**Figure 13.**
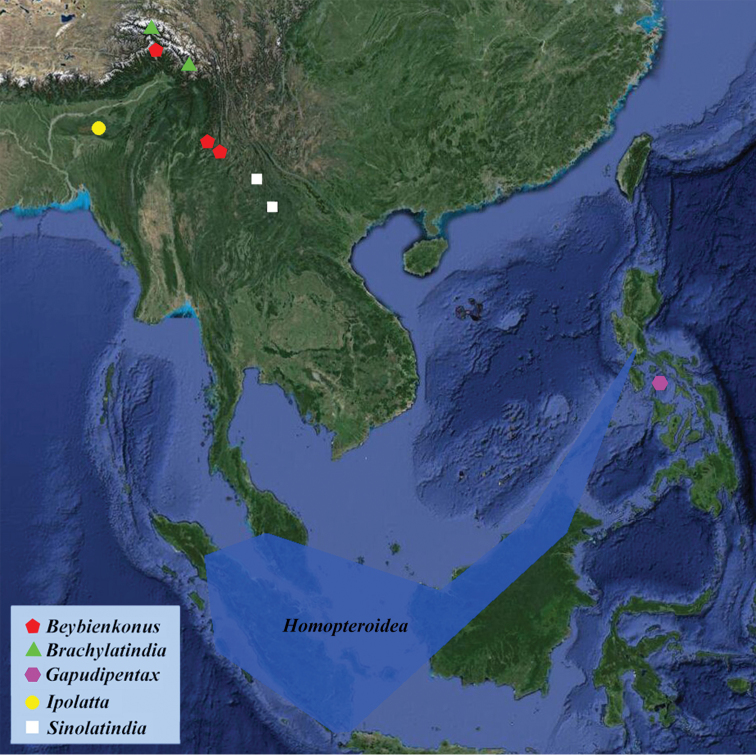
Distribution map of Latindiinae from Asia.

China (Yunnan and Tibet) (Fig. 13).

#### Remarks.

Bey-Bienko (1957) described *Ctenoneura acuticerca* based on two females from Yunnan, China. From the original description, *C. acuticerca* is characterised by the smooth pronotum, absence of intercalary vein and arolia, triangular supra-anal plate with emarginate, valvular subgenital plate, and cerci with a large spine apically. Bey-Bienko (1957) himself had indicated *C. acuticerca* is related to *Ctenoneura aberrans* Hanitsch, 1928. However, *C. aberrans* had been moved to genus *Homopteroidea* since this species is quite different from *Ctenoneura* (Roth 1995a). Later, Qiu et al. (2017) doubted *C. acuticerca* Bey-Bienko, 1957 to be a *Ctenoneura* species according to the absent intercalary vein and arolia, and the female *Ctenoneura* was found to be apterous. However, due to no specimens of *Ctenoneura acuticerca* being available, the problem remained unsolved.

Recently we obtained abundant living individuals of *Ctenoneura acuticerca* from Yingjiang, Yunnan. These roaches were captured from the same locality in the rotten woods. We noticed that this species displays polymorphism. Most individuals are brachypterous, while individuals were very rarely macropterous in the material we examined (Fig. 12B). We compared the brachypterous and macropterous individuals both by morphological features and the COI sequences (one brachypterous male, one brachypterous female and one macropterous female were sequenced, GenBank access numbers MN116497, MN116498 and MN116500, respectively). Both results showed that the brachypterous and the macropterous individuals are conspecific: 1) morphologically, the brachypterous individuals and the macropterous individuals show no differences but in the shape of pronotum and the length of tegmina and wings; and 2) the divergence of COI sequences between the brachypterous male and the macropterous female is 0%, and the divergence between the brachypterous female and the macropterous female is 0.2%. Thus, we confirmed the brachypterous and the macropterous individuals are the same species. Meanwhile, we also sequenced one of the Tibetan specimens by COI (GenBank access number MN116495), and found the divergence between the Tibetan specimen and the Yunnan specimen is only 4.3%–4.4%. Thus, we can confirm that the Tibet individuals are conspecific with the Yunnan individuals.

After a carefully study of *Ctenoneura acuticerca*, we readily confirmed that this species should be excluded from the genus *Ctenoneura* by the winged female, the complex male genitalia with genital hook, and the simplified venation without intercalary vein; and it does not belong to any of the other genera in Corydiidae. We herein establish genus *Beybienkonus* gen. nov. to accommodate *C. acuticerca*. Thus, *Beybienkonus acuticercus* (Bey-Bienko, 1957), comb. nov. is proposed.

## Discussion

In Asia, Latindiinae genera were poorly recorded. Princis (1963) listed only three genera (*Homopteroidea*, *Ipolatta*, *Ctenoneura*) in Latindiidae (now Latindiinae). Only *Ctenoneura* was recently studied and proved to be different from Latindiinae (Qiu et al. 2017), while the other two should be kept as members of Latindiinae.

*Homopteroidea* Shelford currently contains eight species, all of which are restricted to Southeast Asia (Fig. 13). We examined the *Homopteroidea* collection of OUM and consulted former papers (Hanitsch 1929; Roth 1995a; Roth 1995b; Qiu et al. 2016). *Homopteroidea* is proved to belong to Latindiinae by the small and wide apart eyes, simplified venation, the dense fringe-like spinules on the hind margin of front femur, the large white macula at medial supra-anal plate, simplified subgenital plate of male, the complex male genitalia, and the longitudinal incision at subgenital plate of female (valved). This genus is unique among the Latindiinae for the “presutural vein” in tegmina and distinct transparent “presutural zone” in right tegmen (Hanitsch 1929; Roth 1995a; Roth 1995b). However, one “aberrant” species, *Homopteroidea aberrans* (Hanitsch, 1928), has no separate presutural vein or hyaline presutural zone, which therefore requires further study to confirm its status.

*Ipolatta* Karny only contains one species, *via. Ipolatta paradoxa* Karny, 1914, the type specimen is reported from Assam (Karny 1914) (Fig. 13). This genus is characterised by the strongly transverse head (with truncated vertex), discoid and large pronotum (hind margin truncated), horny and veinless tegmina (which exceed the abdomen), and shortened wings. Its supra-anal plate is transverse, and subgenital plate is described as “profunde fissa (= deeply split)”. The head shape of *Ipolatta* resembles that of *Latindia*, *Sinolatindia* and *Gapudipentax*; the character in the subgenital plate indicates that the holotype is a female and is identical to the characters of female Latindiinae. Thus we consider *Ipolatta* as a Latindiinae genus. Nevertheless, this genus is only known from the original description, and its real identity needs further confirmation.

Qiu et al. (2017) also mentioned that *Ctenoneura gigantea* Roth, 1993 was “aberrant” in *Ctenoneura*. This species was described based on one none-abdomen individual from Perak, Malaysia (Roth 1993). The wing venation exhibited in Roth (1993) is in general the Latindiinae type, so we would determine this species to be a Latindiinae. Lacking specimens to study, it may be a new genus, but for now its status remains unsolved.

### Checklist of Latindiinae from Asia

***Homopteroidea* Shelford, 1906**

*Homopteroidea biramiata* Roth, 1995 Indonesia; Malaysia

*Homopteroidea brachyptera* Roth, 1995 Indonesia

*Homopteroidea maculata* Hanitsch, 1929 Indonesia; Malaysia; Philippine

*Homopteroidea minor* Hanitsch, 1933 Malaysia; Indonesia

*Homopteroidea nigra* Shelford, 1906 Malaysia; Indonesia

*Homopteroidea nodipennis* (Karny, 1926) Malaysia; Indonesia

*Homopteroidea shelfordi* Hanitsch, 1925 Malaysia; Indonesia

*Homopteroidea aberrans* (Hanitsch, 1928) Indonesia; Malaysia

***Ipolatta* Karny, 1914**

*Ipolatta paradoxa* Karny, 1914 India (Assam)

***Sinolatindia* Qiu, Che et Wang, 2016**

*Sinolatindia petila* Qiu, Che & Wang, 2016 China (Yunnan)

***Gapudipentax* Lucañas, 2018**

*Gapudipentax guiting* Lucañas, 2018 Philippines (Sibuyan)

***Brachylatindia* Qiu, Wang & Che, gen. nov.**

*Brachylatindia xui* Qiu, Wang & Che, sp. nov. China (Tibet)

***Beybienkonus* Qiu, Wang & Che, gen. nov.**

*Beybienkonus acuticercus* (Bey-Bienko, 1957), comb. nov. China (Yunnan, Tibet)

### Key to the known genera of Latindiinae from Asia

**Table:** 

1	Right tegmen usually with a hyaline presutural zone	*** Homopteroidea ***
–	Right tegmen without hyaline presutural zone	**2**
2	Tegmina veinless, or with indistinct venation	**3**
–	Tegmina with distinct venation	**4**
3	Tegmina horny, veinless	*** Ipolatta ***
–	Tegmina somewhat hyaline, venation absent in male, indistinct in female	*** Gapudipentax ***
4	Arolia absent, male without tergal modification	**5**
–	Arolia present, male with tergal modification	*** Brachylatindia ***
5	Body large, smooth, tarsal claws simple, apex of cerci with a distinct long spine	*** Beybienkonus ***
–	Body small, pubescent, tarsal claws serrated, apex of cerci without a long spine	*** Sinolatindia ***

## Supplementary Material

XML Treatment for Brachylatindia

XML Treatment for Brachylatindia xui

XML Treatment for Brachylatindia

XML Treatment for Beybienkonus

XML Treatment for Beybienkonus acuticercus
